# Ventricular Tachycardia as the Initial Manifestation of COVID-19-Related Myocarditis: A Case Report

**DOI:** 10.7759/cureus.103270

**Published:** 2026-02-09

**Authors:** Lebbar Samy, Youssef Daoudi, Sara Hafid, Fatimazahra Merzouk, El Ghali Mohamed Benouna

**Affiliations:** 1 Cardiovascular Medicine, Mohammed VI International University Hospital, Bouskoura, MAR; 2 Cardiology, Cheikh Khalifa Ibn Zaid International University Hospital, Casablanca, MAR; 3 Cardiology, Mohammed VI International University Hospital, Bouskoura, MAR; 4 Cardiology, Cheikh Khalifa International University Hospital, Mohammed VI University of Health and Sciences (UM6SS), Casablanca, MAR; 5 Cardiology, Mohammed VI International University Hospital, Mohammed VI University of Health Sciences (UM6SS), Casablanca, MAR

**Keywords:** adult cardiology, arrhythmia, covid-19, medical icu, ventricular tachycardia (vt) storm

## Abstract

We report the case of a 53-year-old previously healthy male who presented with sudden-onset palpitations, dizziness, and profuse sweating. Electrocardiography revealed a sustained monomorphic ventricular tachycardia (VT) requiring immediate direct current cardioversion and intravenous amiodarone due to hemodynamic instability. Initial workup, including cardiac biomarkers, echocardiography, and coronary angiography, showed no structural heart disease or coronary obstruction. Cardiac MRI demonstrated circumferential myocardial involvement consistent with myocarditis. Serology confirmed recent SARS-CoV-2 infection, though the patient remained asymptomatic for respiratory symptoms. The patient was managed with amiodarone and colchicine, with no VT recurrence during follow-up. This case highlights an unusual cardiac presentation of COVID-19 and underscores the importance of considering myocarditis in patients presenting with ventricular arrhythmias, even in the absence of typical infectious symptoms.

## Introduction

While the respiratory manifestations of SARS-CoV-2 infection have been widely documented, cardiovascular complications, particularly arrhythmic events, are increasingly recognized and may present in atypical forms [1,2]. Among these, ventricular tachycardia (VT) remains a rare but potentially life-threatening initial presentation, especially in individuals without prior cardiac disease. Myocardial involvement in COVID-19 may arise through several distinct mechanisms, including direct viral entry via angiotensin-converting enzyme 2 (ACE2) receptors, microvascular injury, cytokine-mediated inflammation, and autoimmune responses [3,4]. Unlike other forms of viral myocarditis, which often follow a post-infectious immune-mediated course, COVID-19-related myocarditis may occur during or shortly after the acute phase, sometimes in the absence of systemic symptoms.

We report the case of a previously healthy patient who developed sustained monomorphic VT as the first and sole manifestation of COVID-19-related myocarditis, without respiratory or constitutional symptoms. This case underscores the importance of considering SARS-CoV-2 infection in the differential diagnosis of unexplained ventricular arrhythmias and highlights the diagnostic and therapeutic complexity of managing myocarditis-related VT in the COVID-19 era.

## Case presentation

A 53-year-old man with no prior medical history presented to the emergency department with sudden-onset palpitations, dizziness, and profuse sweating. He denied chest pain, dyspnea, fever, or other systemic symptoms. On examination, he appeared diaphoretic and anxious. His blood pressure was 80/50 mmHg, his heart rate was approximately 210 beats per minute, and he had cool extremities with signs of hypoperfusion.

**Figure 1:**
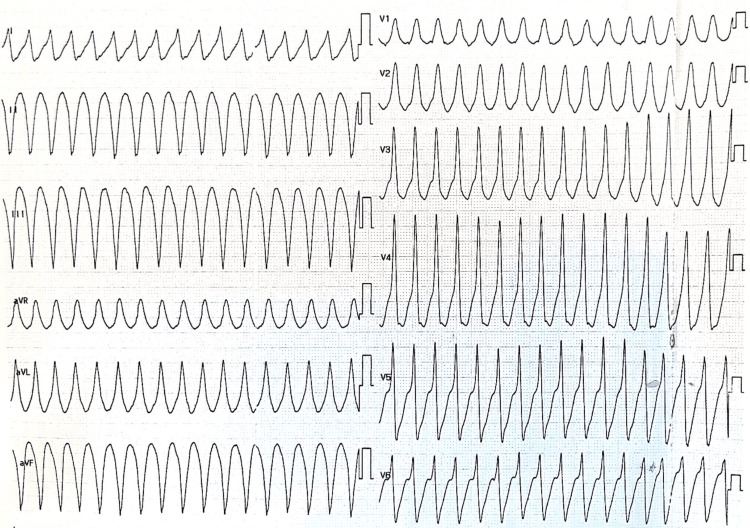
12-lead electrocardiogram on admission showing sustained monomorphic ventricular tachycardia with a wide QRS complex and regular rhythm at ~210 beats/minute, consistent with a ventricular origin. Immediate electrical cardioversion was performed due to hemodynamic instability.

A 12-lead electrocardiogram (ECG) revealed sustained monomorphic VT with a wide QRS complex, right bundle branch block morphology, and a superior axis, suggestive of an inferolateral origin (Figure 1). No capture or fusion beats were observed.

Given the hemodynamic instability, the patient underwent immediate synchronized direct current cardioversion, which restored sinus rhythm. He was subsequently started on an intravenous amiodarone infusion (150 mg loading dose followed by 1 mg/minute for six hours, then 0.5 mg/minute for 18 hours), followed by oral amiodarone (200 mg/day) upon stabilization.

Initial laboratory investigations showed normal serum electrolytes, negative high-sensitivity troponin-T, normal thyroid function tests, and a negative toxicology screen. Inflammatory markers revealed a mildly elevated C-reactive protein of 14 mg/L and erythrocyte sedimentation rate of 28 mm/hour. SARS-CoV-2 polymerase chain reaction (PCR) was negative at admission, while SARS-CoV-2 IgG antibodies were positive. Viral serologies (cytomegalovirus, Epstein-Barr virus, human immunodeficiency virus, hepatitis B/C) and autoimmune markers (antinuclear antibody, anti-neutrophil cytoplasmic antibody) were negative (Table 1).

**Table 1:** Laboratory investigations at admission.

Test	Result	Normal range	Interpretation
Sodium (Na⁺)	Within normal limits	135–145 mmol/L	Normal; excludes hyponatremia as trigger
Potassium (K⁺)	Within normal limits	3.5–5.0 mmol/L	No electrolyte imbalance contributing to arrhythmia
High-sensitivity troponin T	Negative	<14 ng/L	No myocardial infarction or acute injury detected
Thyroid-stimulating hormone	Normal	0.4–4.0 mIU/L	Rules out thyrotoxicosis as a pro-arrhythmic condition
Toxicology screen	Negative	—	No stimulant or substance-related arrhythmogenic cause
C-reactive protein	14 mg/L	<5 mg/L	Mild inflammation
Erythrocyte sedimentation rate	28 mm/hour	<20 mm/hour	Elevated inflammatory response
SARS-CoV-2 PCR	Negative	—	No active infection at admission
SARS-CoV-2 IgG antibodies	Positive	Negative (or non-reactive)	Indicates recent COVID-19 infection; supports viral cause
Viral/Autoimmune serologies	Negative	—	Excludes other systemic causes of myocarditis

**Figure 2:**
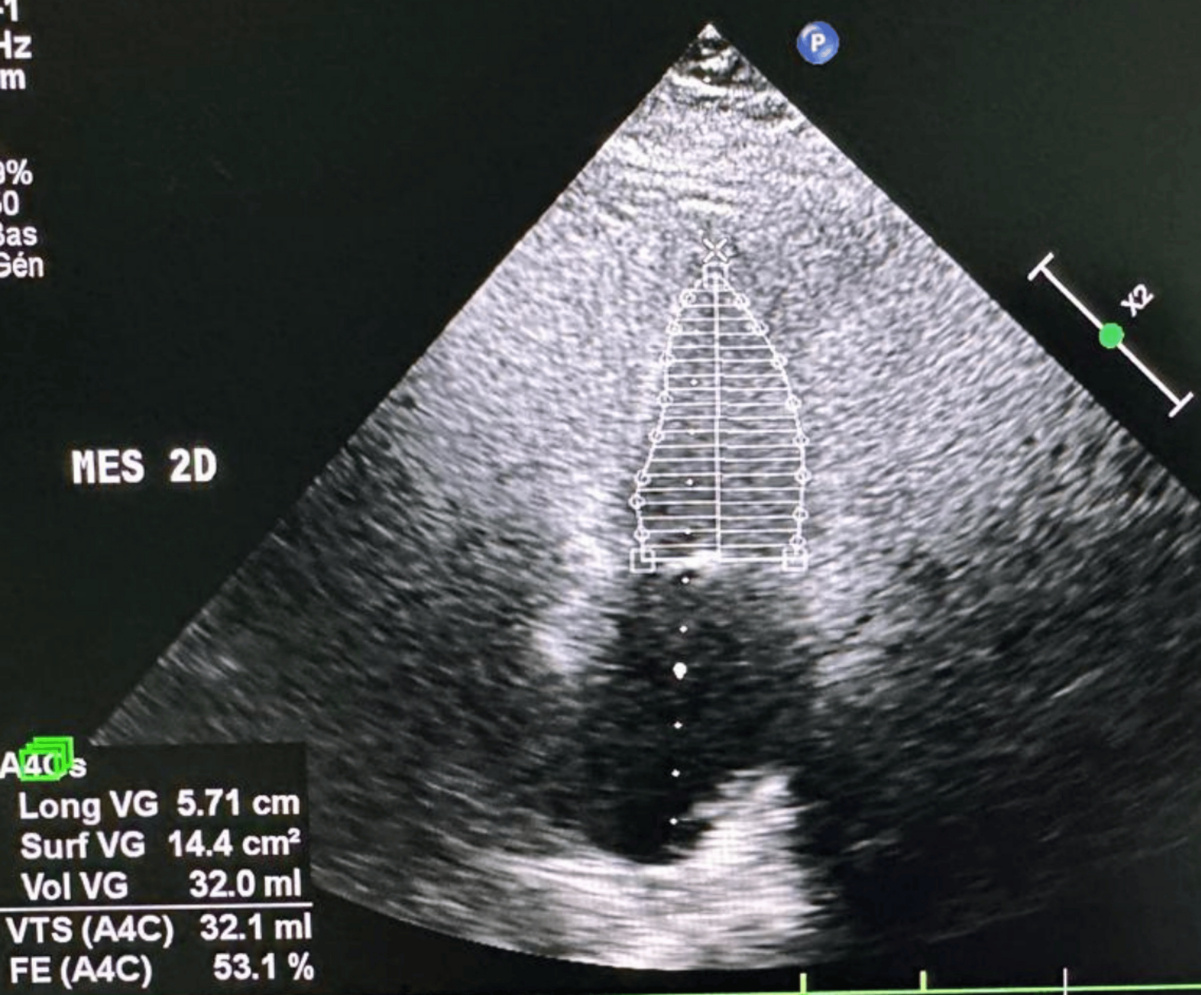
Transthoracic echocardiography. Apical two-chamber view demonstrating normal biventricular systolic function with a preserved left ventricular ejection fraction.

**Figure 3:**
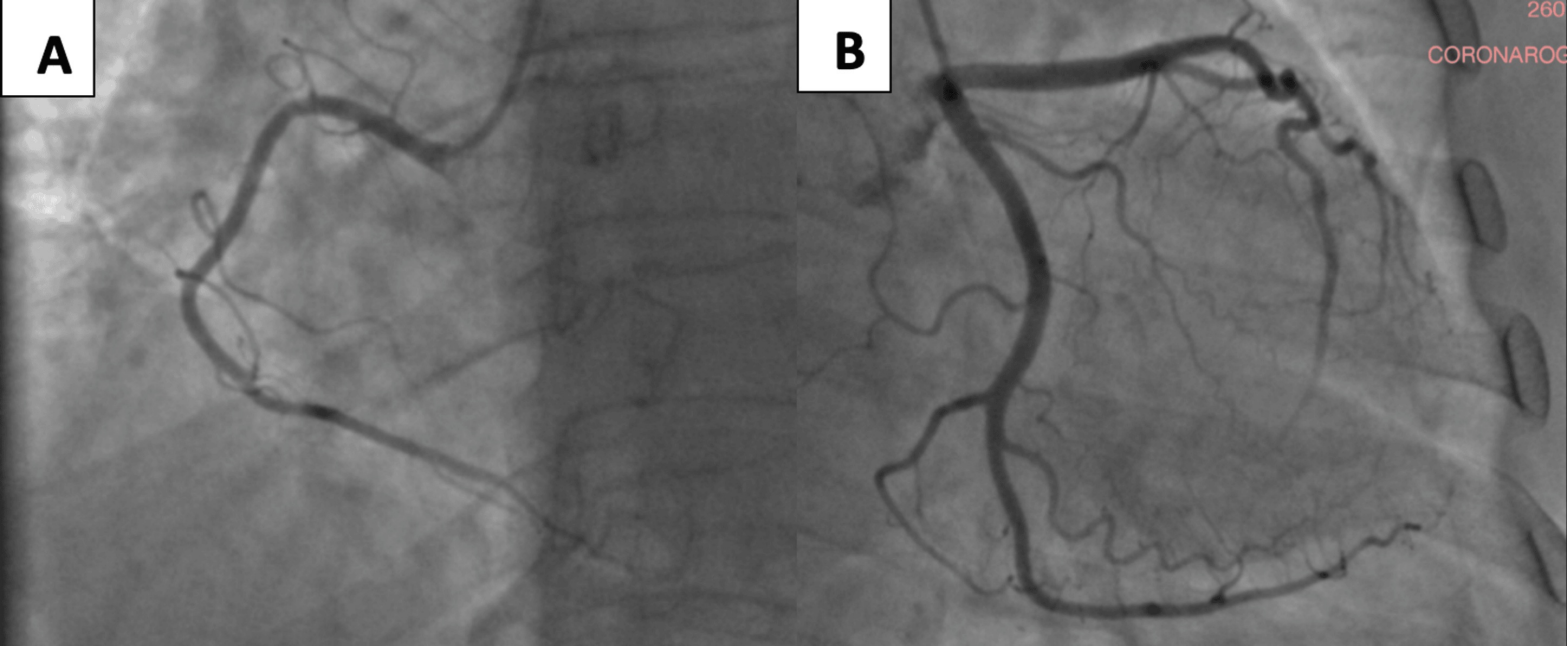
Coronary angiography showing normal coronary anatomy. (A) Right coronary artery without evidence of stenosis. (B) Left coronary artery, including the left anterior descending and left circumflex branches, also free of obstructive lesions.

Transthoracic echocardiography revealed normal left and right ventricular function, with a preserved left ventricular ejection fraction (LVEF = 57%) (Figure 2), no valvular abnormalities, and no regional wall motion abnormalities. Coronary angiography showed a normal coronary anatomy (Figure 3).

**Figure 4:**
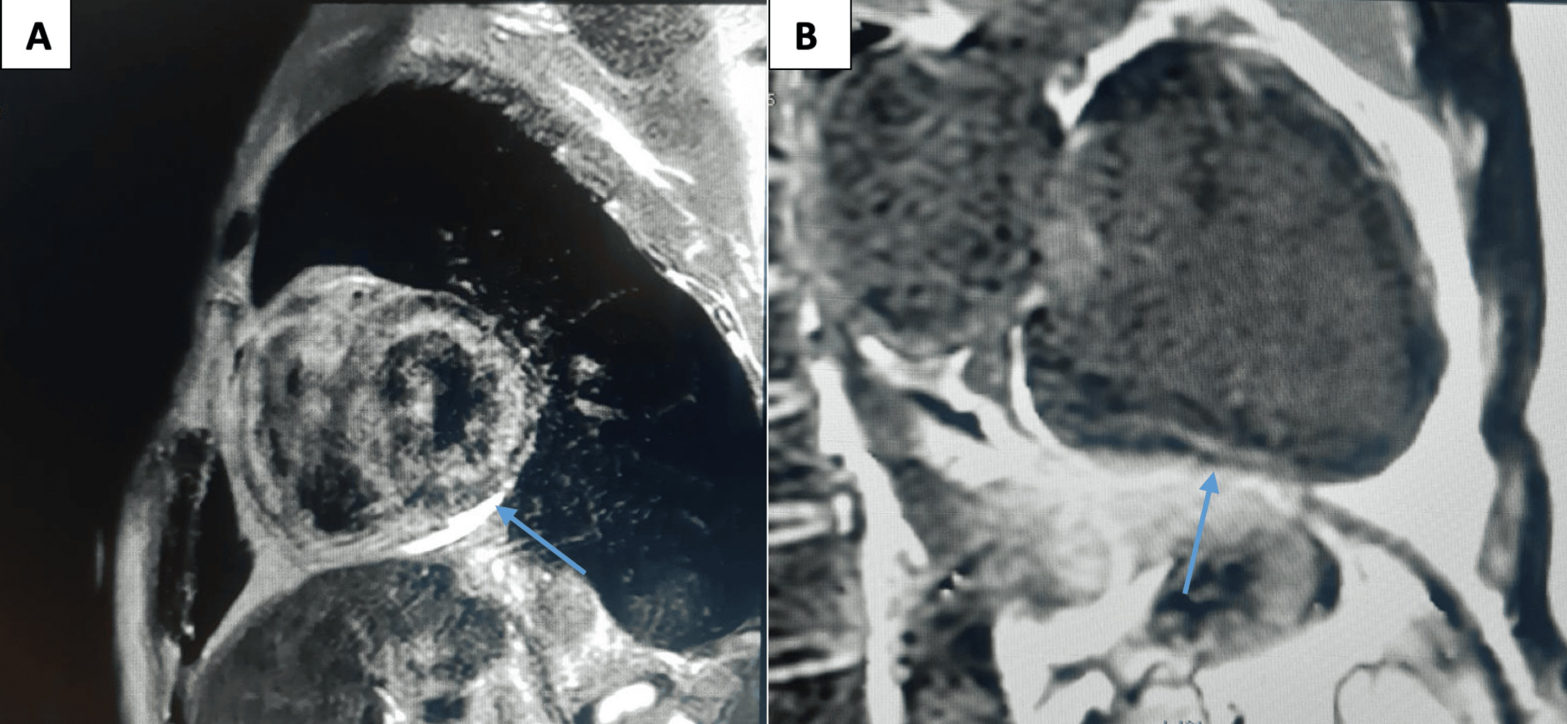
Cardiovascular MRI of the patient showing signs of myocarditis. (A) Short-axis T2-short-tau inversion recovery image showing high signal intensity in the lateral wall (blue arrow), consistent with myocardial edema. (B) Phase-sensitive inversion recovery sequence in long-axis view highlighting subepicardial late gadolinium enhancement in the inferolateral wall (blue arrow), consistent with non-ischemic myocardial injury.

Cardiac magnetic resonance imaging (CMR), performed five days after the VT episode, showed circumferential myocardial edema with subepicardial late gadolinium enhancement involving the lateral and inferolateral walls, consistent with acute myocarditis. Native T1 and T2 mapping were elevated (T1: 1,150 ms, T2: 62 ms). Right and left ventricular systolic function were preserved (LVEF, 57%; right ventricular ejection fraction, 60%) (Figure 4).

The patient had no prior symptoms suggestive of COVID-19, nor had he been tested previously. He was managed conservatively with oral amiodarone (200 mg/day) and colchicine (0.5 mg twice daily for three months) to reduce myocardial inflammation. No further episodes of VT occurred during hospitalization. He was discharged in stable condition and scheduled for structured follow-up, including clinical evaluation and 24-hour Holter monitoring at one, three, and six months.

## Discussion

This case highlights a rare but clinically important presentation of COVID-19: sustained monomorphic VT as the initial manifestation of COVID-19-related myocarditis in a previously healthy individual with no prior cardiovascular history. While the predominant clinical profile of SARS-CoV-2 infection involves respiratory symptoms, cardiovascular involvement, including myocarditis, arrhythmias, thromboembolic events, and myocardial injury, is increasingly recognized [1].

COVID-19 myocarditis may occur through distinct mechanisms, including direct viral entry into cardiomyocytes via ACE2 receptors, immune-mediated injury, and cytokine-induced inflammation [1,2]. Autopsy studies have demonstrated both viral particles and inflammatory infiltrates in myocardial tissue, supporting a direct pathogenic mechanism [3]. Compared to other viral myocarditis etiologies, which often follow a delayed immune-mediated course, COVID-19 myocarditis may present earlier and sometimes in the absence of respiratory symptoms.

Ventricular arrhythmias, including VT and ventricular fibrillation (VF), are generally observed in critically ill COVID-19 patients with elevated biomarkers, structural heart disease, or severe hypoxia [4,5]. In contrast, our patient presented with preserved biventricular function, normal coronary angiography, no elevated troponin, and no overt systemic signs of infection, making this case particularly notable.

CMR, performed five days after the arrhythmic event, revealed myocardial edema and subepicardial late gadolinium enhancement in the lateral wall, consistent with acute myocarditis per the updated Lake Louise criteria. Mapping values were elevated (T1: 1,150 ms, T2: 62 ms), further supporting active myocardial inflammation [6].

Although atrial arrhythmias are more frequently reported in COVID-19, accounting for up to 81.8% of arrhythmic events in large cohorts, ventricular arrhythmias remain rare (estimated incidence 1.2-3.6%) and are associated with a higher risk of sudden cardiac death [7]. This case stands out for its presentation of sustained VT as the sole clinical manifestation of COVID-19 myocarditis, without respiratory symptoms or biomarker elevation.

Management included acute antiarrhythmic therapy with amiodarone and anti-inflammatory treatment with colchicine (0.5 mg BID for three months). While colchicine use in myocarditis is not yet standardized, its efficacy in pericarditis and preliminary data in viral myocarditis support its off-label use [8].

The patient’s SARS-CoV-2 PCR was negative, but IgG serology was positive at admission, consistent with a recent asymptomatic infection. Although we recognize the limitation of inferring causality from serology alone, the exclusion of other infectious and autoimmune causes, in combination with a compatible CMR pattern, supports the diagnosis of COVID-19-associated myocarditis.

No endomyocardial biopsy was performed due to preserved ejection fraction, absence of heart failure, and favorable response to therapy, in line with European Society of Cardiology/American Heart Association recommendations. Differential diagnoses such as sarcoidosis, arrhythmogenic cardiomyopathy, and channelopathies (e.g., Brugada syndrome, long QT) were excluded based on ECG, CMR, clinical history, and absence of genetic predisposition.

The patient was followed over six months with 24-hour Holter monitoring at one, three, and six months. No arrhythmic recurrences occurred. Implantable cardioverter-defibrillator implantation or electrophysiology study was not pursued due to the reversible, inflammatory nature of the disease, absence of recurrent VT, and normalization of cardiac function.

This case underscores the importance of maintaining a high index of suspicion for myocarditis in patients presenting with new-onset VT, especially in the COVID-19 era, even in the absence of respiratory symptoms or biomarker elevation. We suggest that unexplained ventricular arrhythmias in patients with recent or possible SARS-CoV-2 exposure should prompt serological testing and CMR evaluation [9].

## Conclusions

This case highlights the potential for SARS-CoV-2 infection to trigger life-threatening ventricular arrhythmias through myocardial inflammation, even in patients without respiratory symptoms or prior cardiac history. The diagnosis of COVID-19-related myocarditis was supported by CMR, meeting the updated Lake Louise criteria, and positive SARS-CoV-2 IgG serology, in the absence of other viral or autoimmune causes. Although PCR was negative and no biopsy was performed, the clinical, imaging, and serological correlation supported the diagnosis. Prompt recognition and management of sustained VT, combined with targeted antiarrhythmic therapy (amiodarone) and anti-inflammatory treatment (colchicine), led to a favorable outcome without recurrence during six months of follow-up. Clinicians should maintain a broad differential diagnosis when evaluating new-onset arrhythmias in patients with normal coronary anatomy and no known cardiac disease. In the current epidemiological context, COVID-19-related myocarditis should be considered, even in asymptomatic individuals, when CMR shows a non-ischemic pattern of injury. Early use of CMR and structured follow-up are essential to guide management and avoid unnecessary device implantation in reversible causes. This case adds to the growing body of literature on atypical cardiac presentations of COVID-19 and reinforces the importance of post-viral myocarditis surveillance.
